# Branched-Chain Amino Acids Have Equivalent Effects to Other Essential Amino Acids on Lifespan and Aging-Related Traits in *Drosophila*

**DOI:** 10.1093/gerona/glz080

**Published:** 2019-03-20

**Authors:** Paula Juricic, Sebastian Grönke, Linda Partridge

**Affiliations:** 1 Max Planck Institute for Biology of Ageing, and Department of Biological Mechanisms of Ageing, Cologne, Germany; 2 Institute of Healthy Ageing, and Department of Genetics, Evolution and Environment, UCL, London, UK

**Keywords:** Intestinal dysplasia, Lipid metabolism, Fecundity

## Abstract

Branched-chain amino acids (BCAAs) have been suggested to be particularly potent activators of Target of Rapamycin (TOR) signaling. Moreover, increased circulating BCAAs are associated with higher risk of insulin resistance and diabetes in both mice and humans, and with increased mortality in mice. However, it remains unknown if BCAAs play a more prominent role in longevity than do other essential amino acids (EAAs). To test for a more prominent role of BCAAs in lifespan and related traits in *Drosophila*, we restricted either BCAAs or a control group of three other EAAs, threonine, histidine and lysine (THK). BCAA restriction induced compensatory feeding, lipid accumulation, stress resistance and amelioration of age-related gut pathology. It also extended lifespan in a dietary-nitrogen-dependent manner. Importantly, the control restriction of THK had similar effects on these phenotypes. Our control diet was designed to have every EAA equally limiting for growth and reproduction, and our findings therefore suggest that the level of the most limiting EAAs in the diet, rather than the specific EAAs that are limiting, determines the response of these phenotypes to EAA restriction.

Dietary restriction (DR) extends lifespan and protects against many age-associated pathologies in an array of model organisms (1–4). The health and lifespan benefits of DR have long been attributed to reduced intake of calories. However, mounting evidence points toward protein and even single amino acid restrictions, without changes in caloric content of the diet, as responsible for the health benefits of DR (5–7). In *Drosophila*, restricting dietary protein extends lifespan, whereas carbohydrate and lipid restriction have little or no effect on survival (7). Moreover, low dietary protein to carbohydrate ratio extends lifespan in flies (8) and mice (6). Essential amino acids (EAAs) play a central role in lifespan extension by protein restriction, because add-back of EAAs, but not non-essential amino acids (NEAAs), abolishes lifespan extension by DR in both, flies (5) and mice (9). In humans, several epidemiological studies have found a positive correlation between protein-rich diets and early mortality (10,11), and have highlighted the importance of age-dependent changes in dietary protein requirements (11). However, the identity of the EAAs that mediate lifespan extension by protein restriction remains elusive.

The branched-chain amino acids (BCAAs), leucine, isoleucine, and valine, have been suggested to be of particular importance for health and longevity because of their prominent role in the activation of the Target of Rapamycin (TOR) pathway (12). However, studies investigating the role of dietary BCAAs in aging and health have yielded contradictory results. For instance, BCAA supplementation improved survival and cardiac and skeletal muscle mitochondrial biogenesis in middle-aged male mice (13). Similarly, knock-down of BCAA transferase-1, the enzyme responsible for the first step of BCAA catabolism, resulted in increased BCAA levels and extended lifespan in Caenorhabditis *elegans* (14). In contrast, elevated circulating BCAAs have been associated with insulin resistance, diabetes and obesity in mice (6) and humans (15,16) and with increased mortality in mice (6). In line with these findings, reducing dietary BCAAs, but not other EAAs, improved glucose homeostasis in mice (17). These opposing effects of BCAAs are currently not well understood and could result in part from different EAA composition of the controls diets used in these studies.

To address if dietary BCAA have a particularly prominent role in aging, we compared the effects of restriction of BCAAs or of a control group of three other EAAs, thus far not reported to affect lifespan, threonine, histidine and lysine (THK), on lifespan, feeding behavior, stress resistance and age-related gut pathology of fruit flies. To ensure that our comparison of the effect of amino acid identity was not confounded with the effects of degree of restriction, we used for a control diet a holidic medium with an exome-matched amino acid ratio optimized for fly growth, reproduction and lifespan (18). In this medium every EAA is limiting for growth and reproduction, meaning that restriction of any amino acid would result in growth and fecundity reduction. We made equal restrictions of the group of three BCAAs or of THK. We found that BCAA restriction induced compensatory feeding, lipid accumulation, stress resistance and amelioration of age-related gut pathology. BCAA restriction extended lifespan in a dietary-nitrogen-dependent manner. Importantly, restriction of the three other EAAs, THK, had similar effects on these phenotypes, suggesting that the level of the most limiting EAAs, rather than amino acid identity, shapes the response of these phenotypes to EAA restriction.

## Methods

### Fly Husbandry

For all experiments, a wild type white *Dahomey* (w^*Dah*^) fly strain that naturally carries the endosymbiontic bacterium *Wolbachia pipientis* was used. Stocks were kept at 25°C on a 12:12 hours light:dark cycle, at constant humidity (65%). Crosses were set-up in cages containing grape juice plates with a small amount of yeast paste and embryos were collected over 24 hours period by washing the plate with PBS. Embryos were squirted into bottles containing sugar/yeast/agar diet (yeast supplier was MP Biomedicals LLC, Cat no: 903312) at ~20 μl per bottle to achieve standard larval density (20 μl embryos = ~300 eclosed flies per bottle). Eclosed adult flies (females and males) were collected over an 18-hour period and allowed to mate for 48 hours in fresh sugar/yeast/agar diet containing bottles. After this period female flies were collected and assigned to one of the experimental diets.

### Holidic Media for *Drosophila*

Holidic media were prepared according to (19) with slight modifications (Supplementary Tables S1 and S2). Briefly, agar, sucrose and amino acids with low solubility (L-leucine, L-isoleucine and L-tyrosine) were added to the milliQ water containing solutions of metal ions and cholesterol. The mixture was autoclaved for 15 minutes at 120°C, using a Mediaclave 10 (Integra Biosciences), followed by addition of filter-sterilized acetate buffer and solutions of the remaining amino acids, vitamins, nucleotides, inositol, choline, and preservatives. For all experiments, an exome-matched amino acid ratio (18) was used. For BCAA- and THK-restricted diets, BCAAs/THK were reduced by 85% compared to the level present in the control diet (Supplementary Tables S1 and S2). To compensate for the reduction of total dietary nitrogen, all other amino acids were proportionately increased, thus all diets were iso-nitrogenous and iso-caloric.

### Lifespan and Fecundity Assay

Flies were transferred into fresh vials every 2–3 days, and the number of dead flies was scored. For fecundity assays, eggs were collected over 16- to 24-hour periods multiple times during the first 4 weeks of adult life and the number of eggs was counted using a hand counter.

### Proboscis Extension Assay

Five flies were transferred into vials containing fresh food and proboscis extension rates were measured the following morning 2 hours after the lights were switched on, according to a previously described protocol (20), with slight modifications. Feeding events were scored every 10 minutes for 10 times in total. Feeding rates of experimental groups are expressed as the proportion of feeding flies (sum of scored feeding events / total number of feeding opportunities, where total number of feeding opportunities = number of flies in vial × number of vials in the group × number of observations). For each experimental group, eight biological replicates were used. Proboscis extension experiments were performed under blinded conditions.

### Starvation Assay

One hundred flies were aged for 2 weeks on experimental diets at a density of 20 flies per vial and afterward transferred to the starvation food containing 1% agarose (Ultra Pure, Invitrogen) in ddH_2_O. Number of dead flies was scored three times per day.

### Imaging and Scoring Gut Dysplasia

Dissected guts were fixed in 4% formaldehyde for 30 minutes and mounted using mounting medium containing 1.5 μg/mL DAPI (Vectashield, H1200). DAPI was imaged using a confocal microscope (Leica SP5-X). For each condition, 6–14 guts were imaged in the area proximal to the proventriculus, and three adjacent images for each gut were taken. Area affected by tumors was measured using the measure function in ImageJ, and the average proportion of the affected area for each gut was calculated. For scoring gut dysplasia, areas that had large absorptive cells, enterocytes, aligned to form a single layer epithelium with evenly spaced nuclei were scored as non-pathological. Epithelium with several layers of nuclei, formation of small nuclear “nests”, containing unpolarized cells clustering apically and leading to loss of epithelial organization, were scored as dysplastic regions. Data acquisition and analysis was done under blinded conditions.

### Triacylglyceride Assay

Flies were aged for 10 days on experimental diets, snap-frozen in liquid nitrogen and used for triacylglyceride (TAG) content measurements according to (21). Briefly, five flies in five replicates were homogenized in 1 mL 0.05% Tween20, using the Fastprep-24 system (MP Biomedicals). The homogenate was incubated at 70°C for 5 minutes followed by centrifugation at 1400 rpm for 5 min. The supernatant was diluted 1:10, and 50 μl was used to measure baseline absorbance at 540 nm. 200 μl of Thermo Infinity Triglycerides solution (ThermoScientific), prewarmed to 37°C, was added to 50 μl of supernatant and absorbance was measured at 540 nm. Absolute TAG concentration was determined using a TAG standard (Cayman Chemical).

### Protein Extraction From Fly Tissues

Twenty heads were homogenized in 2× Laemmli loading sample buffer (100 mM Tris pH 6.8, 20% glycerol, 4% SDS) using a hand homogenizer. Extracts were cleared by centrifugation and protein content determined by using the BCA Protein Assay Kit (ThermoFisher). 5% β-mercaptoethanol was added and samples were boiled for 5 min.

### Western Blotting

Equal amounts of proteins (10 μg) were loaded and separated using pre-stained SDS-PAGE gels (Bio-Rad), proteins were wet-transferred and the membrane was blocked using 5% nonfat dry milk powder / TBST at room temperature (RT) for 1 h. Blocked membrane was incubated with primary antibodies overnight (ON) at 4°C. Afterward, membrane was washed, appropriate HRP-coupled secondary antibody was applied (ThermoFisher). For signal development, ECL Select Western Blotting Detection Reagent (GE Healthcare) was applied and images were captured using ChemiDocImager (Bio-Rad). Antibodies used in the study were phopho-S6K (CST #9209) and S6K (22).

### Statistical Analysis

Statistical analysis was performed using Graphpad Prism and JMP and individual statistical tests described in corresponding figure legends.

## Results

### Feeding and Metabolic Phenotypes are Similarly Affected by Restriction of BCAAs or of THK

**Figure 1.**
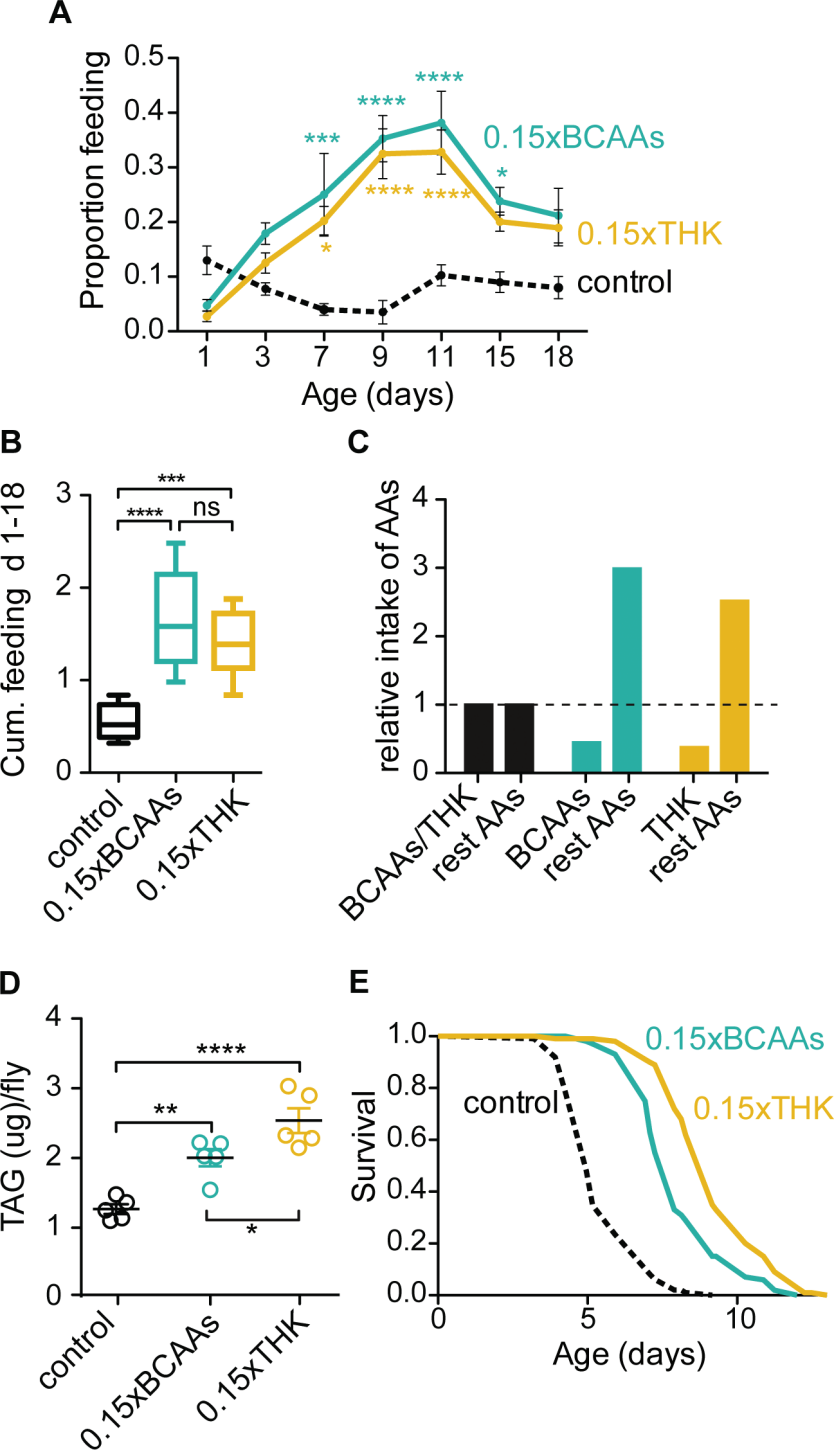
Restriction of branched-chain amino acids (BCAAs) or of threonine, histidine and lysine (THK) induced compensatory feeding, increased lipid content and starvation resistance. (**A**–**B**) BCAA (*p* = .0001) and THK restriction (*p* = .001) induced compensatory feeding to similar degree (*p* > .5). There was significant interaction between diet and age (*p* < .0001). *N* = 8. Two-way analysis of variance (ANOVA) followed by Bonferroni’s post-test (**A**) and One-way ANOVA followed by Bonferroni’s post-test (**B**). (**C**) Relative intake of BCAAs, THK and rest of the amino acids in BCAA- and THK-restricted diets compared to control diet calculated from proboscis extension assay. (**D**) Restriction of BCAAs (*p* < .01) and THK (*p* < .0001) induced lipid accumulation in 14-day-old flies, with THK restriction having stronger effect compared to BCAA restriction (*p* < .05). *N* = 5. One-Way ANOVA followed by Bonferroni’s test. *p < .05, **p < .01, ***p < .001, ****p < .0001. (**E)** Pretreatment with a diet restricted in BCAAs or THK for 14 days increased survival under starvation, and restriction of THK increased survival under starvation to a slightly greater degree than did restriction of BCAAs (control v 0.15xBCAAs: *p* = 4.34 × 10^−27^; control v 0.15xTHK: *p* = 6.19 × 10^−41^; 0.15xBCAAs v 0.15xTHK: *p* = 3.09 × 10^−07^). *N* = 100. Experiments were performed three times (Supplementary Figure S2). Log-rank test and Cox proportional hazard analysis (Supplementary Table S3).

Dietary protein and amino acid levels are important determinants of fertility and lifespan, and animals direct their food selection to precisely control protein intake (23). Reduced dietary protein, therefore, leads to an increase in food consumption, the so-called protein leverage effect (24). Moreover, dietary amino acid ratios can affect feeding rates in flies, with a suboptimal ratio of amino acids for growth and reproduction resulting in increased food intake (18). To assess whether BCAA restriction had a stronger effect on feeding rate than did restriction of THK, we used diets with 85% BCAA restriction (0.15xBCAAs) or with an equivalently reduced proportion of THK (0.15xTHK) compared to the control diet (Supplementary Figure S1A and B). To compensate for reduced total nitrogen levels, a proxy for amino acid abundance, in the BCAA/THK drop-down diets, which alone may affect feeding rates and lifespan (18), we proportionally increased all other amino acids to the same total level as in the control diet (Supplementary Figure S1A and B). Thus, all diets were iso-caloric and iso-nitrogenous. To assess feeding rates we performed proboscis extension assays (20) in *Drosophila* females. Surprisingly, BCAA and THK restriction increased feeding behavior to a similar degree (Figure 1A and B). Despite the increase in feeding rates under BCAA and THK restriction, combining feeding data with amino acid concentration in the diets showed that flies ingested lower amounts of the restricted amino acids on the restricted diets compared to control flies and over-consumed the amino acids that were not restricted (Figure 1C).

Increased feeding rates led to increased ingestion of the other dietary nutrients in addition to the non-restricted amino acids, including lipids and carbohydrates, potentially driving obesity and lipid accumulation. We, therefore, investigated if restriction of BCAAs or of THK also affected lipid (triglycerides, TAG) levels in the flies. Both, BCAA- and THK- restriction elevated TAG levels, with the restriction of THK having slightly stronger effects compared to the restriction of BCAAs (Figure 1D).

Increased energy reserves in form of triglycerides can increase survival under starvation (25). We, therefore, tested if pretreatment of flies with BCAA-/THK-restricted diets improved starvation resistance. In line with our findings that restricting BCAAs/THK elevated triglyceride levels and that THK restriction increased lipid storage to a slightly higher degree (Figure 1D), these interventions also increased survival under starvation, again with THK restriction having a slightly stronger effect compared to BCAA restriction (Figure 1E and Supplementary Figure S2A and B). To compare the degree of increased starvation resistance by BCAA and THK restriction, we performed Cox Proportional Hazard (CPH) analysis, which confirmed that THK restriction increased starvation resistance even further than restriction of BCAAs (Supplementary Table S3). Together, these data indicate that restriction of BCAAs or of THK caused similar effects on feeding and lipid metabolism that were, if anything, stronger with THK restriction.

### Restriction of BCAAs Extends *Drosophila* Lifespan to the Same Degree as THK Restriction

**Figure 2.**
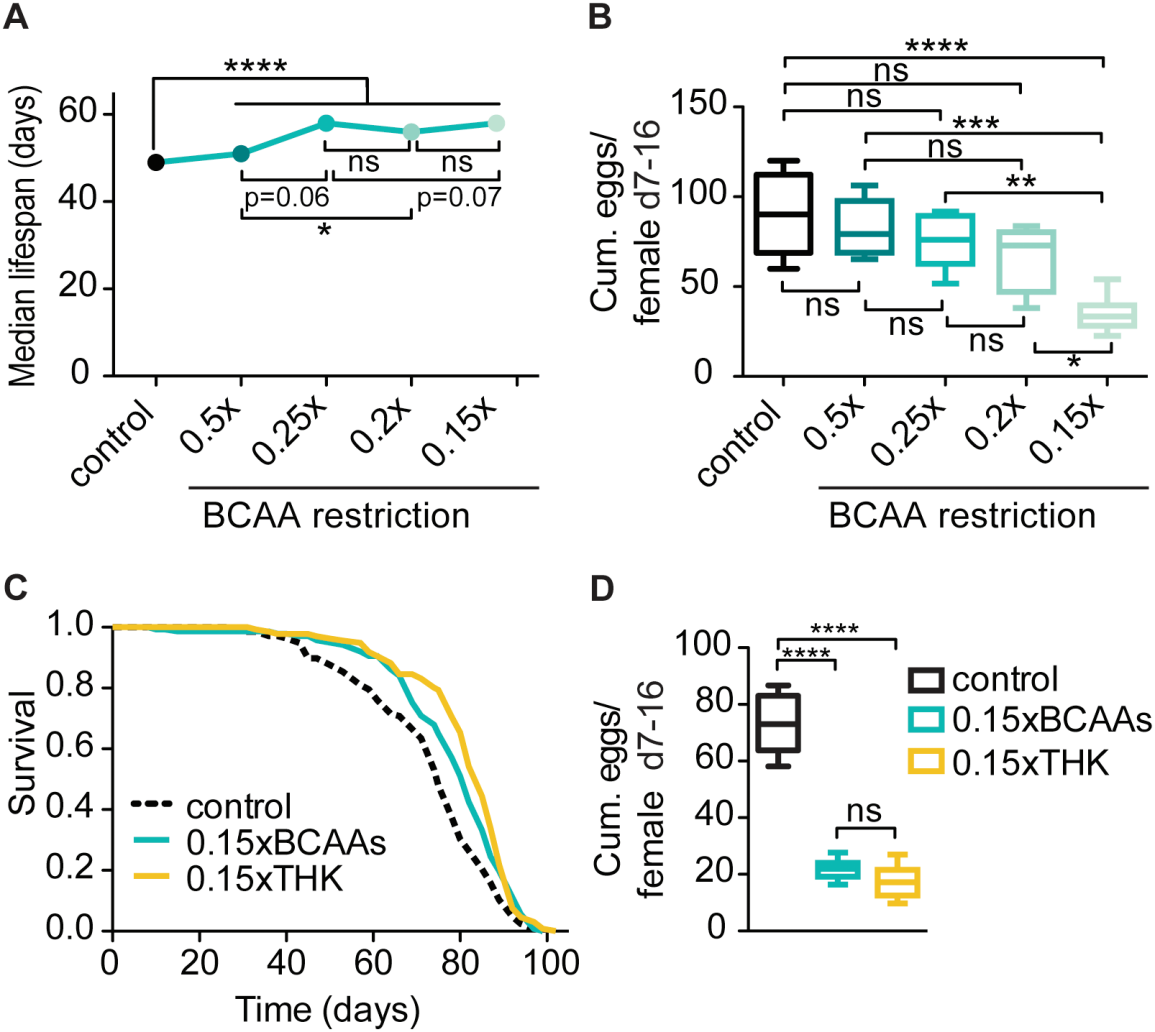
Restriction of branched-chain amino acids (BCAAs) extended fly lifespan and reduced fecundity to a similar degree as threonine, histidine and lysine (THK) restriction. (**A**) Restriction of BCAAs extended lifespan in a dose-dependent manner. Restricting BCAAs from 0.5xBCAAs to 0.25xBCAAs did not increase survival further, whereas decreasing it to 0.2xBCAAs and 0.15xBCAAs additionally extended lifespan. *N* = 200. Log-rank test. *p < .05, ****p < .0001. (**B**) BCAA restriction reduced cumulative egg-laying measured from Day 7–16 only when BCAAs were restricted to 0.15x of their concentration in the control diet. *N* = 6. One-way analysis of variance (ANOVA) followed by Bonferroni’s test. **p < .01, ***p < .001, ****p < .0001. (**C**) Restriction of BCAAs (*p* = .002) or THK (*p* = 8.35 × 10^−07^) extended lifespan to the same extent (*p* = .08). *N* = 200. Experiments were performed three times (Supplementary Figure S3). Log-rank test and Cox proportional hazard analysis (Supplementary Table S4A–C). (**D**) Restriction of BCAAs (*p* < .0001) or THK (*p* < .0001) reduced cumulative egg-laying measured from Day 7–14 to the same degree (*p* > .05). *N* = 10. One-way ANOVA followed by Bonferroni’s test.

Based on the finding that plasma levels of BCAAs negatively correlate with lifespan in mice (6), we postulated that restricting dietary BCAAs would extend fly lifespan. To test this idea, we kept flies on diets with varying degrees of BCAA restriction ranging from 50% (0.5xBCAAs) to 85% (0.15xBCAAs) and measured their lifespan and fecundity. 50% BCAA restriction resulted in a small, but significant, lifespan extension without affecting fecundity (Figure 2A and B). Further restriction of BCAAs by 80% and 85% resulted in an additional increase in lifespan, whereas fecundity was only significantly decreased upon restriction of 85% BCAA. The uncoupling of lifespan and fecundity upon mild BCAA restriction suggests that reduced fecundity is not the main cause of lifespan extension by BCAA restriction.

To assess whether BCAAs play an especially prominent role in determining lifespan and fecundity, we compared the effects of 85% restriction THK on lifespan and egg-laying. Interestingly, restriction of THK reduced egg-laying and extended lifespan to the same level as BCAA restriction (Figure 2C and D and Supplementary Figure S3A and B), and CPH analysis confirmed that the magnitude of lifespan extension by BCAA and THK restriction was not significantly different (Supplementary Table S4A–C). These results indicate that the degree of amino acid restriction, rather than the identity of amino acids, is important for lifespan extension by restriction of EAAs.

**Figure 3.**
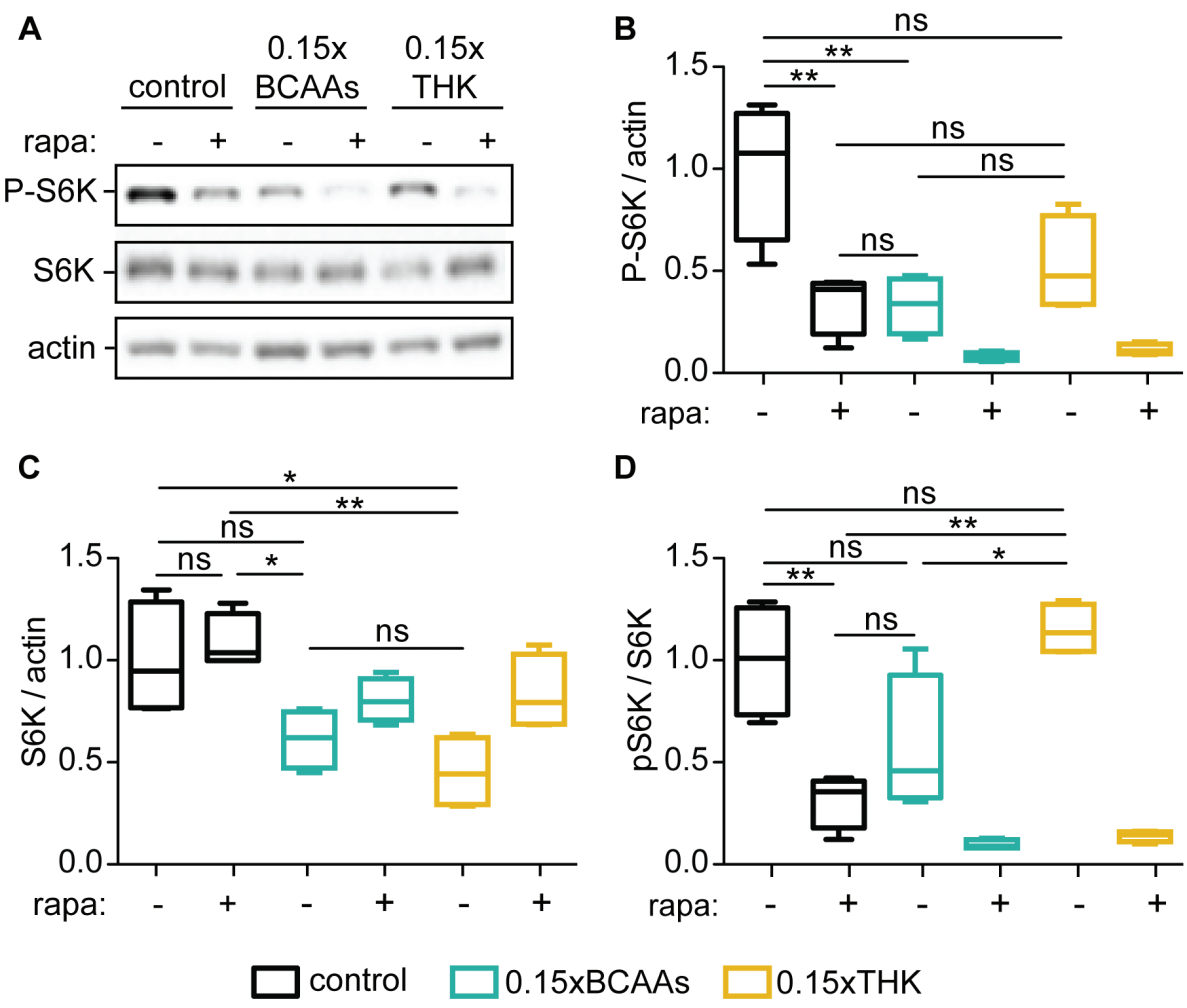
Branched-chain amino acids (BCAAs) and threonine, histidine and lysine (THK) restrictions reduced phosphorylated and total S6K levels to similar degree. (**A**–**B**) BCAA restriction reduced the levels of phosphorylated S6K to a similar degree as rapamycin treatment, and THK restriction showed similar trend towards reduced phosphorylated S6K levels. (**A**–**C**) THK restriction reduced total S6K levels and BCAA restriction showed a similar trend, whereas rapamycin had no effect on total S6K levels. (**D**) BCAA and THK restriction had no effect on the ratio of phosphorylated to total S6K. *N* = 4. *p < .05, **p < .01, ***p < .001. One-way analysis of variance (ANOVA) followed by Bonferroni’s test.

Since BCAAs were suggested to be specifically potent activators of the TOR signaling pathway (12), we assessed whether BCAA restriction had more prominent effect on TOR activity compared to THK restriction. We measured phosphorylation of S6K, a direct downstream target of TORC1, in flies treated with BCAA-/THK-restricted diets for 2 weeks. Rapamycin treatment on the respective diets was included as positive control. We found that BCAA restriction reduced the levels of phosphorylated S6K to a similar degree as rapamycin treatment on the control diet, and although not significant THK restriction caused a similar reduction of phosphorylated S6K levels (Figure 3A and B). Interestingly, while the levels of total S6K were unaffected by rapamycin treatment, THK and BCAA restriction both reduced total S6K levels (Figure 3A–C). Rapamycin reduced the ratio of phosphorylated to total S6K, whereas BCAA and THK restriction had no significant effect on their ratio, most likely due to the concurrent downregulation of pS6K and total S6K levels (Figure 2A–D). Noteworthy, the strongest downregulation of pS6K levels was observed on the amino acid restricted diets plus rapamycin. This might indicate combinatorial effects of diet and drug treatment, but might also be explained by the higher food uptake of these flies, which would also result in higher uptake of rapamycin. In summary, these results suggest that BCAA and THK restrictions had an equal effect on TOR activity and reduced both total and phosphorylated S6K.

### BCAA- and THK-Restriction Ameliorate Age-Related Intestinal Pathology to the Same Degree

**Figure 4.**
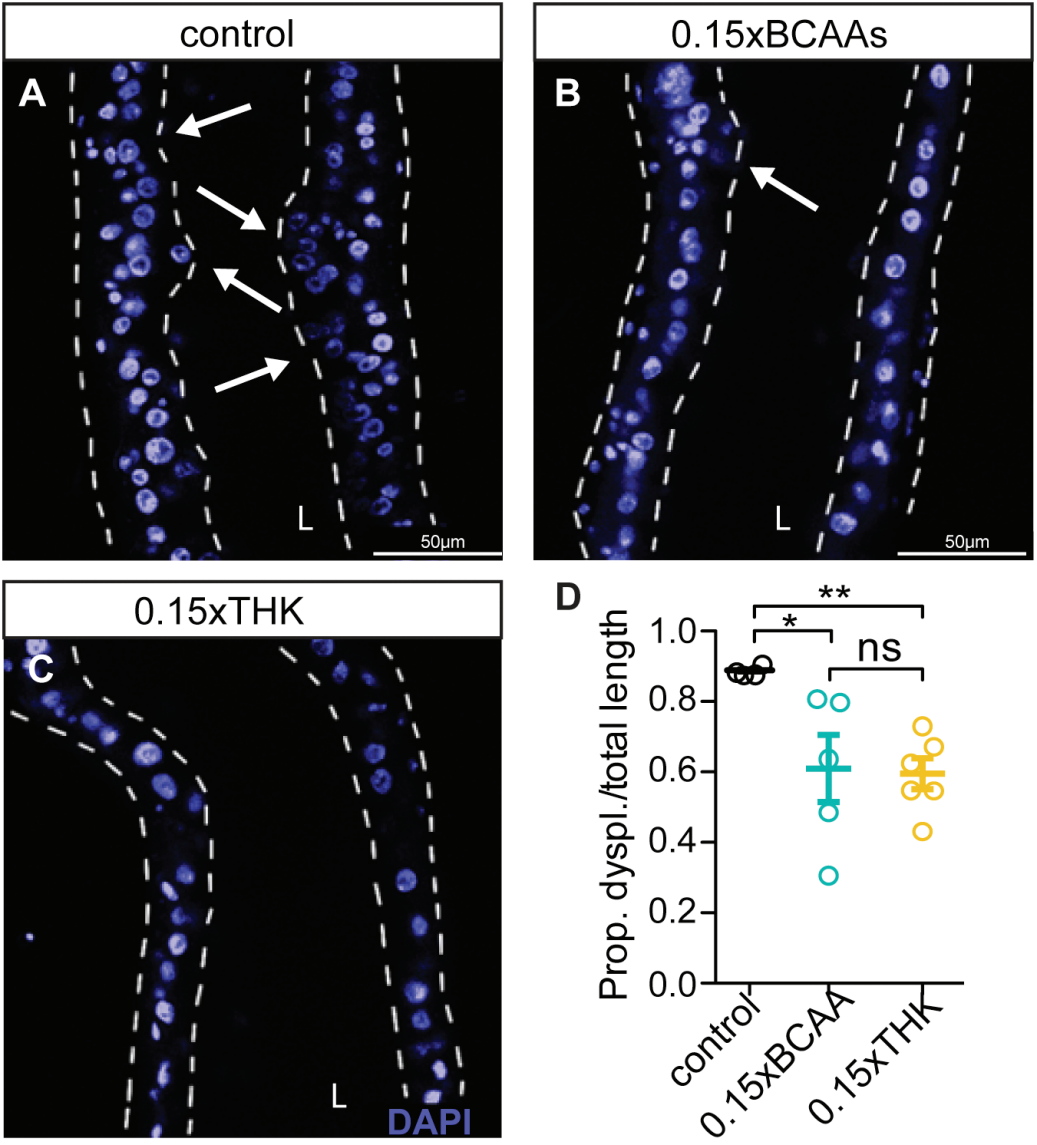
Restriction of branched-chain amino acids (BCAAs) and of threonine, histidine and lysine (THK) ameliorated age-related intestinal pathology to the same level. (**A–D**) BCAA (**B**) and THK restriction (**C**) reduced age-related intestinal dysplasia in 60-day-old females. For scoring intestinal dysplasia: regions with enterocytes aligned to form a single layer epithelium with evenly spaced nuclei were scored as non-pathological. Epithelium with several layers of nuclei, formation of small nuclear “nests,” containing unpolarized cells clustering apically, were scored as dysplastic regions. L-lumen. *N* = 5. *p < .05, **p < .01, ***p < .001. One-way analysis of variance (ANOVA) followed by Bonferroni’s test.

Restricting yeast (protein-rich) in a diet ameliorates age-related intestinal pathologies (26) and preserving intestinal homeostasis is associated with longer lifespan (26–28). We therefore tested whether restriction of BCAAs or THK might also confer beneficial effects on the aging intestine. Restriction of either BCAAs (Figure 4B) or of THK (Figure 4C) ameliorated age-related intestinal pathology and tumor formation compared to the control diet (Figure 4A), and to a similar degree to each other (Figure 4D). Together, these results show that restriction of BCAAs or of THK increases feeding rates and lipid accumulation, extends lifespan and ameliorates age-related intestinal pathology to the same degree, suggesting that the response of these phenotypes to EAA restrictions is determined solely by the degree of EAA restriction and not by the type of restricted amino acids.

### BCAA Restriction Extends Lifespan in Dietary-Amino-Acid-Dependent Manner

**Figure 5.**
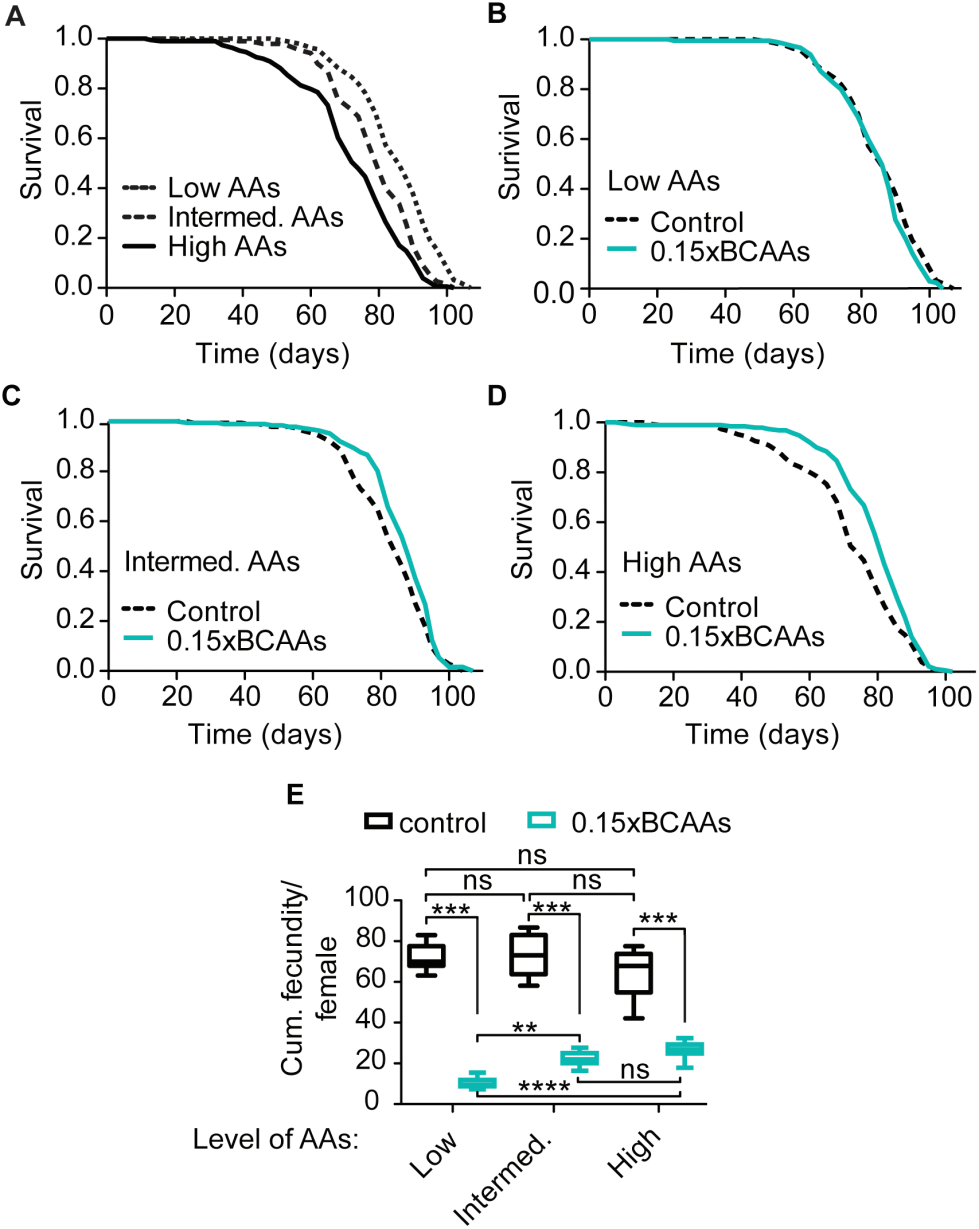
Lifespan extension and reduced fecundity by branched-chain amino acids (BCAAs) restriction depended on dietary amino acid concentration. (**A**) Reducing dietary amino acid concentration from high to intermediate (*p* < .0001) and from intermediate to low extended lifespan (*p* < .001). (**B**) BCAA restriction on low amino acid condition did not extend lifespan (*p* = .099). (**C**–**D**) Restriction of BCAAs under conditions of intermediate (**C,***p* = .016) and high (**D,***p* < .00012) dietary amino acid concentrations extended lifespan. Log-rank test and Cox proportional hazard analysis (Supplementary Table S5). (**E**) Restriction of BCAAs reduced fecundity measured from Day 7–14 on low, intermediate and high-amino-acid diets (*p* < .0001). Restricting BCAAs on low amino acid condition had stronger effect on fecundity compared to BCAAs restriction on intermediate and high amino acid conditions. There was significant interaction between amino acid restriction condition and amino acid level (*p* < .0001). *N* = 10; *p < .05, **p < .01, ***p < .001, ****p < .0001. Two-way analysis of variance (ANOVA) followed by Bonferroni’s post-test.

Having established that BCAA and THK restrictions similarly affected all phenotypes we measured thus far, we continued by focusing on BCAA restriction for further experiments. Because dietary protein is an important determinant of lifespan (5,6,11) we next investigated whether the effect of EAA restriction on lifespan was dependent on dietary amino acid abundance. We combined BCAA restriction with diets containing varying levels of amino acids, low (11 g/L food), intermediate (22 g/L food) and high (33 g/L food), and found that, as previously reported (5–7,11), reducing dietary amino acid content extended lifespan (Figure 5A). Interestingly, BCAA restriction had no effect on lifespan on a low-amino-acid diet (Figure 5B), but extended lifespan of flies fed intermediate- and high-amino-acid diets (Figure 5C and D), suggesting an overlapping mechanism for lifespan extension by reduced total dietary amino acid content and reduced proportion of BCAAs. CPH analysis confirmed that reducing dietary amino acids from high to intermediate and low concentrations, reduced mortality and that BCAA restriction affected survival differently depending on dietary amino acid concentration (Supplementary Table S5). Next, we assessed if BCAA restriction affected egg-lying in a dietary-amino-acid-dependent manner and found that restriction of BCAAs reduced fecundity under all dietary amino acid conditions. However, the effect was strongest on the low amino acid diet (Figure 5E), which supports the idea that fecundity is more sensitive to amino acid limitation at low protein levels.

Increased survival with reduced BCAAs was associated with increased resistance to starvation (Figure 1E), and we, therefore, investigated whether BCAA restriction also affected starvation resistance differently at different total amino acid concentrations. BCAA restriction increased starvation resistance at all dietary amino acid concentrations. However, CPH analysis showed that the effect was least at low amino acid level and stronger at intermediate and high amino acid levels (Supplementary Figure S4A–C, Supplementary Table S6). Together, these findings suggest that BCAA limitation and the low-amino-acid diet may act through overlapping mechanisms to increase lifespan and through partially overlapping mechanisms to increase starvation resistance.

## Discussion

In mice and humans, reduced dietary protein content is associated with increased lifespan (6,11). BCAAs have gained considerable attention in the context of aging, as they were suggested to be more potent activators of TOR signaling compared to other amino acids (12). However, to date, reports about the effects of BCAAs on aging have produced inconsistent results (6,13). Moreover, it remains unknown if BCAAs affect life history traits differently from other EAAs. Here we show that restriction of BCAAs or a set of three amino acids, THK, that have not been previously implicated in regulation of longevity, increase food intake, lipid content, stress resistance, extend lifespan and reduce age-related intestinal dysplasia in *Drosophila* in a similar manner. We demonstrate that lifespan extension by BCAA restriction depends on the dietary amino acid content, providing a possible explanation for contradicting results obtained by previous studies.

Elevated circulating BCAAs were associated with increased mortality in mice (6), and in line with this, we demonstrated here that reducing dietary BCAAs extended lifespan in flies. In contrast, several other studies showed beneficial effects of increased BCAAs for lifespan (13,14), that can possibly be explained by several factors. First, positive effects of BCAAs on lifespan could be explained by weight loss induced by BCAA supplementation in specific conditions as increased body mass is associated with many prevalent human diseases (23). For example, local administration of leucine into the brain suppresses food intake in mice (29,30) and dietary BCAA supplementation reduces food intake and body mass of mice fed a high-fat diet (31). Similarly, mice deficient in mitochondrial branched-chain aminotransferase (BCATm), exhibiting increased circulating BCAAs levels, had reduced body mass compared to control mice (32). Second, contrasting results obtained from different studies may be explained by different basal diets, which, as shown here and by others (33), can affect the study outcome. Third, BCAA could improve health through maintenance of autoimmune homeostasis, as BCAAs were shown to increase proliferation and function of Foxp3+ regulatory T (Treg) cells (34), which are important for prevention of autoimmunity (35).

Interestingly, BCAA restriction extended lifespan on intermediate and high total amino acid diets, but not under the low amino acid diet, suggesting that BCAA- and total-amino-acid-restriction extend lifespan through overlapping mechanisms. These data are in contrast with findings that methionine restriction extends lifespan only under low amino acid condition in *Drosophila* (33). This discrepancy could be explained by different basal diets used in the two studies, but also by the amino acid specific responses to total amino acid level variations, as methionine omission was shown to induce transcriptional response distinct from that caused by omission of other amino acids in MCF7 cells (36). However, it highlights the importance of the impact of the baseline diet on study outcome and could explain contrasting results obtained in different studies measuring the same parameters. One such examples is the University of Wisconsin and NIA studies investigating the effects of DR on lifespan and health in rhesus monkeys. While the first study showed that DR extends lifespan in rhesus monkeys, the latter study found an improvement in health without lifespan extension (3,37) and, among other parameters, different baseline diets and source of protein might have contributed to opposing results in the aforementioned studies.

We showed here that decreasing proportion of dietary BCAAs and THK by 85% increased fly feeding rates to the same level, possibly reflecting an attempt to increase fecundity, which has been proposed to be limited by the most limiting amino acid in the diet (18). Our data show that egg-laying is reduced to a similar degree on diets restricted in BCAAs and THK, suggesting that the level of the most limiting amino acid, rather than the type of restricted amino acid, determines fecundity. Similarly, omitting either arginine or isoleucine from a diet decreased fecundity to the same level in flies (19). Reducing the cost of reproduction and reallocating nutrients away from reproduction to somatic maintenance was long held to be responsible for lifespan extension by DR (38,39). We show here that moderate modulations of BCAAs that extend lifespan do not reduce fecundity, which is in line with several studies demonstrating that fecundity and lifespan can be uncoupled (5,18,33,40). Thus, the reallocation hypothesis is not sufficient to explain lifespan extension by EAA restriction.

Despite the major progress in determining lifespan response to dietary amino acid modulations (41), the underlying molecular mechanisms remain elusive. One of the possible mechanisms by which BCAA and THK restrictions extend lifespan is by reducing TORC1 activity, as shown for methionine restriction in flies (33). Similarly, our results indicate that the TOR activity is equally but only mildly reduced by both BCAA and THK restrictions. Downstream of TORC1, several factors might contribute to lifespan extension by BCAA restriction. For instance, downregulation of mRNA translation, through reduced levels of phosphorylated S6K, which we also observed in BCAA and THK-restricted flies, is already implicated in longevity. Mutation in S6K extends lifespan in yeast (42), nematodes (43), flies (44) and mice (45) and directly reducing mRNA translation extends lifespan in yeast (46) and nematodes (47). Another potential mediator of lifespan extension by BCAA restriction is induction of autophagy, which was shown to mediate lifespan extension by rapamycin treatment in flies (22) and by DR in *C. elegans* (48). Moreover, we showed that BCAA- and THK- restriction ameliorated age-related intestinal dysplasia, potentially contributing to increased lifespan (26,27,49).

In summary, we showed here that BCAA restriction extends lifespan, increases stress resistance and ameliorates age-related intestinal pathology to a similar degree as the restriction of three other EAAs, providing an important piece of evidence that the concentration, rather than the type of the most limiting amino acids determines the aforementioned phenotypes. Moreover, we demonstrate the importance of the nutritional context in which such experiments are carried out, offering a possible explanation for disagreement in different studies investigating similar questions. Future studies should address the conservation of these phenotypes in mammals and may contribute to the development of dietary interventions to improve health in humans.

## Funding

The research leading to these results has received funding from the European Research Council under the European Union’s Seventh Framework Programme (FP7/2007–2013)/ ERC grant agreement n° 268739. We acknowledge funding from the Max Planck Society.

## Conflict of Interest

None reported.

## Supplementary Material

glz080_suppl_Supplementary_Materials
