# Supplementary material for: Branched-Chain Amino Acids Have Equivalent Effects to Other Essential Amino Acids on Lifespan and Aging-Related Traits in Drosophila
Source: J Gerontol A Biol Sci Med Sci. 2019 Mar 20;75(1):24–31. doi: 10.1093/gerona/glz080 (PMC6909895; doi:10.1093/gerona/glz080)
Supplement: glz080_suppl_Supplementary_Materials [file glz080_suppl_supplementary_materials.docx]

**Supplementary Materials**

Figure S1 **Amino acid levels in diets with restricted BCAAs (A) and THK (B).**

**
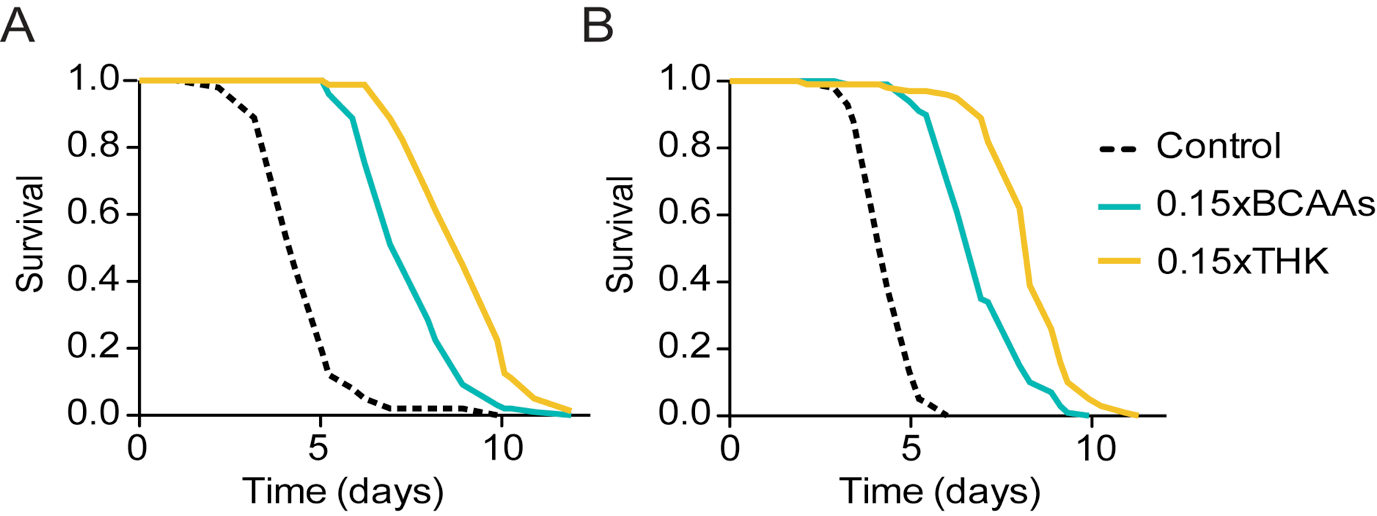
**

Figure S2 **Repeats of experiments investigating the effects of BCAA- and THK- restrictions on starvation resistance. (A - B)** BCAA- and THK- restricted diets increased sur­­­vival under starvation, with THK restriction having slightly stronger effect (**A**; control v 0.15xBCAAs: p=2.30x10^-32^; control v 0.15xTHK: p=1.08x10^-36^; 0.15xBCAAs v 0.15xTHK: p=1.16x10^-09^, **B**; control v 0.15xBCAAs: p=6.35x10^-24^; control v 0.15xTHK: p=5.89x10^-46^; 0.15xBCAAs v 0.15xTHK: p=2.53x10^-13^). N=100. Log-rank test.


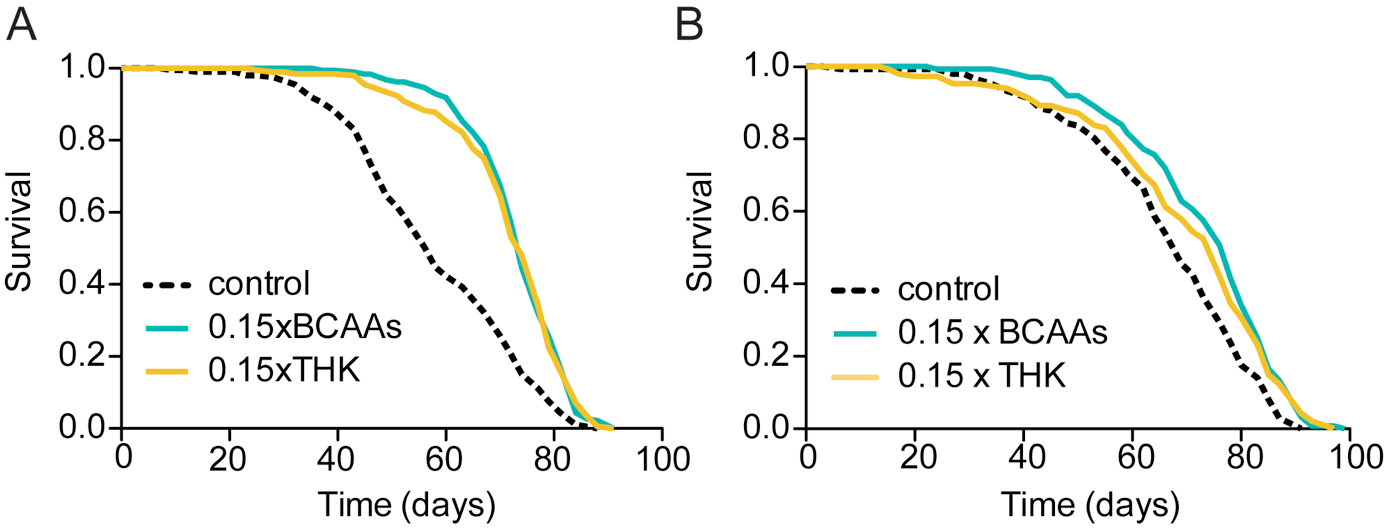


Figure S3 **Repeats of experiment investigating the effects of BCAA- and THK- restrictions on lifespan. (A)** BCAA- (p=1.596 x 10^-18^) and THK- (p=4.50 x 10^-17^) restrictions extended lifespan to the same degree (p=0.76). **(B)** Restriction of BCAAs (p=5.96 x 10^-05^) or THK (p=0.0027) extended lifespan to the same extent (p=0.38). N=200. Log-rank test.

**
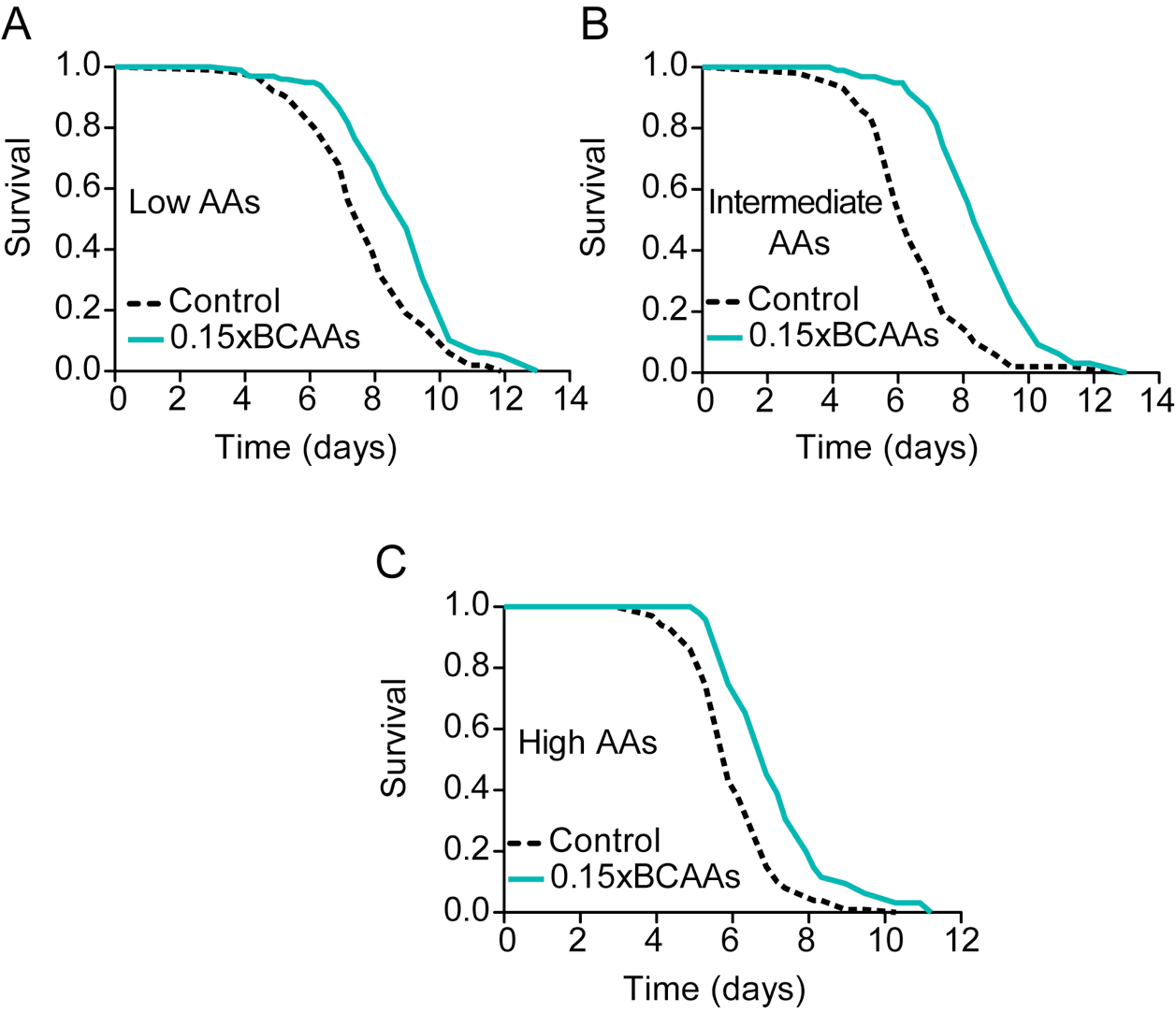
**

Figure S4 **BCAA restriction increases survival upon starvation in amino-acid-level-dependent manner.**

(**A**-**C**) BCAA restriction increased survival under starvation on low (**A**, p=2.11 x 10^-05^), intermediate (**B**, p=1.54 x 10^-14^), and high (**C**, p=4.69 x 10^-08^), amino acid conditions to varying degrees (see Table S6). N=100. Log-rank test and Cox proportional hazard analysis (Table S7).

Table S1 **Holidic media recipes for branched-chain-amino-acid (BCAA)- and threonine, histidine, lysine (THK)- restricted diets.**

|  | **Control** | **0.15xBCAAs** | **0.15xTHK** |  |
| --- | --- | --- | --- | --- |
| total volume (L) | 1 | 1 | 1 | |
| mM Sucrose | 50 | 50 | 50 | |
| mM biologically available N | 200 | 200 | 200 | |
| Isoleucine (g) | 1.12 | 0.2 | 1.28 | |
| Leucine (g) | 2.03 | 0.36 | 2.31 | |
| Tyrosine (g) | 0.93 | 1.09 | 1.06 | |
| Agar (g) | 20 | 20 | 20 | |
| Sucrose (g) | 17.12 | 17.12 | 17.12 | |
| Cholesterol (ml) | 15 | 15 | 15 | |
| Acetate buffer (ml) | 100 | 100 | 100 | |
| CaCl2 (1000x, ml) | 1 | 1 | 1 | |
| MgSO4 (1000x, ml) | 1 | 1 | 1 | |
| CuSO4 (1000x, ml) | 1 | 1 | 1 | |
| FeSO4 (1000x, ml) | 1 | 1 | 1 | |
| MnCl2 (1000x, ml) | 1 | 1 | 1 | |
| ZnSO4 (1000x, ml) | 1 | 1 | 1 | |
| MilliQ water (ml) | 805.09 | 780.63 | 784.94 | |
| Nucleic acids/lipid solution (ml) | 8 | 8 | 8 | |
| **EAA** (ml) | 60.51 | 70.80 | 68.98 | |
| **NEAA** (w/o cys, ml) | 60.51 | 70.80 | 68.98 | |
| Glutamate (ml) | 15.79 | 18.47 | 18.00 | |
| Cystein (ml) | 7.10 | 8.31 | 8.09 | |
| Vitamins (ml) | 21 | 21 | 21 | |
| Folic acid (ml) | 1 | 1 | 1 | |
| Ppropionic acid (ml) | 6 | 6 | 6 | |
| Nipagin (ml) | 15 | 15 | 15 | |

Table S2 **Amino acid content in stock solutions used for holidic media preparation.**

|  | **Essential amino acid** | **g/200ml stock solution** |  | **Non-essential amino acid** | **g/200ml stock solution** | |
| --- | --- | --- | --- | --- | --- | --- |
|  |  |  |  |  |  |  |
| Control | phenylalanine | 3.47 |  | alanine | 3.79 | |
|  | histidine | 2.25 |  | aspartate | 4.03 | |
|  | lysine | 4.69 |  | glycine | 2.64 | |
|  | methionine | 2.07 |  | asparagine | 3.54 | |
|  | arginine | 5.61 |  | proline | 3.36 | |
|  | threonine | 3.81 |  | glutamine | 3.85 | |
|  | valine | 4.13 |  | serine | 4.74 | |
|  | tryptophan | 1.10 |  | **glutamate | 20 | |
|  | isoleucine* |  |  | ***cysteine | 10 | |
|  | leucine* |  |  |  | | |
| 0.15xBCAAs | phenylalanine | 3.47 |  | alanine | | 3.79 |
|  | histidine | 2.25 |  | aspartate | | 4.03 |
|  | lysine | 4.69 |  | glycine | | 2.64 |
|  | methionine | 2.07 |  | asparagine | | 3.54 |
|  | arginine | 5.61 |  | proline | | 3.36 |
|  | threonine | 3.81 |  | glutamine | | 3.85 |
|  | valine | 0.62 |  | serine | | 4.74 |
|  | tryptophan | 1.10 |  | **glutamate | | 20 |
|  | isoleucine* |  |  | ***cysteine | | 10 |
|  | leucine* |  |  |  | | |
| 0.15xTHK | phenylalanine | 3.47 |  | alanine | | 3.79 |
|  | histidine | 0.34 |  | aspartate | | 4.03 |
|  | lysine | 0.70 |  | glycine | | 2.64 |
|  | methionine | 2.07 |  | asparagine | | 3.54 |
|  | arginine | 5.61 |  | proline | | 3.36 |
|  | threonine | 0.57 |  | glutamine | | 3.85 |
|  | valine | 4.13 |  | serine | | 4.74 |
|  | tryptophan | 1.10 |  | **glutamate | | 20 |
|  | isoleucine* |  |  | ***cysteine | | 10 |
|  | leucine* |  |  |  | | |

*isoleucine and leucine were added in solid form during media preparation, due to their low solubility. **glutamate and ***cysteine stock solutions were made separately from the rest of the non-essential amino acids.

Table S3 **Statistical analysis for Figure 1, E**

|  | Survival under starvation: BCAA- v THK-restriction | | | | | |  |  | |  |
| --- | --- | --- | --- | --- | --- | --- | --- | --- | --- | --- |
|  |  |  | | |  |  |  |  | |  |
|  | **p-value (log rank)** |  | | |  |  |  |  | |  |
|  |  | control | | | 0.15xBCAAs | 0.15xTHK |  |  | |  |
|  | median (d) | 4.6 | | | 7.6 | 8.7 |  |  | |  |
|  | mean (d) | 5.5 | | | 8 | 9.5 |  |  | |  |
|  | control |  | | | 4.34E-27 | 6.19E-41 |  |  | |  |
|  | 0.15xBCAAs |  | | |  | 3.10E-07 |  |  | |  |
|  | 0.15xTHK |  | | |  |  |  |  | |  |
|  |  |  | | |  |  |  |  | |  |
|  | **Cox Proportional Hazard (CPH) analysis** | | | | |  |  |  | |  |
|  | Number of events | |  | 298 | | | | |  | |
|  | Number of censoring |  | 0 | | | | |  | |  |
|  | Total number | |  | 298 | | | | |  | |
|  |  | |  | | | |  |  | |  |
|  | **Risk Ratios** |  | | |  |  |  |  | |  |
|  | Level 1 | /Level 2 | | | Risk Ratio | p | Lower 95% | Upper 95% | |  |
|  | control | 0.15xBCAAs | | | 5.4721369 | <0.0001 | 3.9940774 | 7.5018784 | |  |
|  | 0.15xTHK | 0.15xBCAAs | | | 0.5439980 | <0.0001 | 0.4088775 | 0.7233468 | |  |
|  | 0.15xTHK | control | | | 0.0994124 | <0.0001 | 0.0707977 | 0.1393563 | |  |
|  | 0.15xBCAAs | control | | | 0.182744 | <0.0001 | 0.1332999 | 0.2503707 | |  |
|  | 0.15xBCAAs | 0.15xTHK | | | 1.838242 | <0.0001 | 1.3824628 | 2.4457201 | |  |
|  | control | 0.15xTHK | | | 10.059112 | <0.0001 | 7.1758521 | 14.12476 | |  |

Table S4 **Statistical analysis for Figure 2, C and S3, A-B**

|  | | |  |  |  |  |
| --- | --- | --- | --- | --- | --- | --- |
|  | **(A)** Survival: BCAA- v THK-restriction (Figure 2, C) | | |  |  |  |
|  | **p-value (log rank)** |  |  |  |  |  |
|  |  | control | 0.15xBCAAs | 0.15xTHK |  |  |
|  | median (d) | 74.5 | 81.0 | 83.5 |  |  |
|  | mean (d) | 72.7 | 78.4 | 82.2 |  |  |
|  | maximum (d) | 90.5 | 94.0 | 90.5 |  |  |
|  | control |  | 0.00216141 | 8.3567E-07 |  |  |
|  | 0.15xBCAAs |  |  | 0.08418833 |  |  |
|  | 0.15xTHK |  |  |  |  |  |
|  |  |  |  |  |  |  |
|  |  |  |  |  |  |  |
|  | **Cox Proportional Hazard (CPH) analysis** | | |  |  |  |
|  |  |  |  |  |  |  |
|  | Number of events |  | 400 |  |  |  |
|  | Number of censoring |  | 29 |  |  |  |
|  | Total number |  | 429 |  |  |  |
|  |  |  |  |  |  |  |
|  | **Risk Ratios** |  |  |  |  |  |
|  | Level 1 | /Level 2 | Risk Ratio | p | Lower 95% | Upper 95% |
|  | 0.15xBCAAs | control | 0.6970747 | 0.0035 | 0.547042 | 0.8880576 |
|  | 0.15xTHK | control | 0.5718786 | <0.0001 | 0.4488227 | 0.7286144 |
|  | 0.15xTHK | 0.15xBCAAs | 0.8203979 | 0.1095 | 0.6437716 | 1.0456023 |
|  | control | 0.15xBCAAs | 1.4345665 | 0.0035 | 1.1260531 | 1.8280133 |
|  | control | 0.15xTHK | 1.7486229 | <0.0001 | 1.372468 | 2.2280515 |
|  | 0.15xBCAAs | 0.15xTHK | 1.2189207 | 0.1095 | 0.9563865 | 1.553346 |

|  | | |  |  |  |  |
| --- | --- | --- | --- | --- | --- | --- |
|  | **(B)** Survival: BCAA- v THK-restriction (Figure S3, A) | | |  |  |  |
|  | **p-value (log rank)** |  |  |  |  |  |
|  |  | control | 0.15xBCAAs | 0.15xTHK |  |  |
|  | median (d) | 57 | 73 | 73 |  |  |
|  | mean (d) | 58.2 | 73.5 | 72.1 |  |  |
|  | maximum (d) | 80 | 82.5 | 82.5 |  |  |
|  | control |  | 1.5961E-18 | 4.5047E-17 |  |  |
|  | 0.15xBCAAs |  |  | 0.76317577 |  |  |
|  | 0.15xTHK |  |  |  |  |  |
|  |  |  |  |  |  |  |
|  |  |  |  |  |  |  |
|  | **Cox Proportional Hazard (CPH) analysis** | | |  |  |  |
|  |  |  |  |  |  |  |
|  | Number of events |  | 579 |  |  |  |
|  | Number of censoring |  | 31 |  |  |  |
|  | Total number |  | 610 |  |  |  |
|  |  |  |  |  |  |  |
|  | **Risk Ratios** |  |  |  |  |  |
|  | Level 1 | /Level 2 | Risk Ratio | p | Lower 95% | Upper 95% |
|  | 0.15xBCAAs | control | 0.4261747 | <0.0001 | 0.3474345 | 0.5224571 |
|  | 0.15xTHK | control | 0.4398573 | <0.0001 | 0.3599432 | 0.5374654 |
|  | 0.15xTHK | 0.15xBCAAs | 1.0321058 | 0.759 | 0.8434467 | 1.2635472 |
|  | control | 0.15xBCAAs | 2.3464558 | <0.0001 | 1.9140327 | 2.87824 |
|  | control | 0.15xTHK | 2.2734644 | <0.0001 | 1.860585 | 2.7782163 |
|  | 0.15xBCAAs | 0.15xTHK | 0.9688929 | 0.759 | 0.7914228 | 1.185611 |
|  | | |  |  |  |  |
|  | **(C)** Survival: BCAA- v THK-restriction (Figure S3, B) | | |  |  |  |
|  | **p-value (log rank)** |  |  |  |  |  |
|  |  | control | 0.15xBCAAs | 0.15xTHK |  |  |
|  | median (d) | 67.5 | 77.0 | 74.5 |  |  |
|  | mean (d) | 66.6 | 73.7 | 70.2 |  |  |
|  | maximum (d) | 86.0 | 89.0 | 89.0 |  |  |
|  | control |  | 5.96852E-05 | 0.002720692 |  |  |
|  | 0.15xBCAAs |  |  | 0.380793474 |  |  |
|  | 0.15xTHK |  |  |  |  |  |
|  |  |  |  |  |  |  |
|  |  |  |  |  |  |  |
|  | **Cox Proportional Hazard (CPH) analysis** | | |  |  |  |
|  |  |  |  |  |  |  |
|  | Number of events |  | 417 |  |  |  |
|  | Number of censoring |  | 10 |  |  |  |
|  | Total number |  | 427 |  |  |  |
|  |  |  |  |  |  |  |
|  | **Risk Ratios** |  |  |  |  |  |
|  | Level 1 | /Level 2 | Risk Ratio | p | Lower 95% | Upper 95% |
|  | 0.15xBCAAs | control | 0.6546288 | 0.0006 | 0.513632 | 0.833225 |
|  | 0.15xTHK | control | 0.7051267 | 0.0030 | 0.551102 | 0.8864745 |
|  | 0.15xTHK | 0.15xBCAAs | 1.0716404 | 0.5697 | 0.8444228 | 1.3618606 |
|  | control | 0.15xBCAAs | 1.5275832 | 0.0006 | 1.2001561 | 1.9469193 |
|  | control | 0.15xTHK | 1.4254626 | 0.0030 | 1.128064 | 1.8014442 |
|  | 0.15xBCAAs | 0.15xTHK | 0.9331489 | 0.5697 | 0.7342896 | 1.1842409 |

Table S5 **Statistical analysis for Figure 5**

|  |  |  |  |  |  |  |  |
| --- | --- | --- | --- | --- | --- | --- | --- |
|  | Survival: BCAA restriction on varying amino acid concentrations | | | |  |  |  |
|  |  |  |  |  |  |  |  |
|  | **p-value (log rank)** | Low AAs control | LowAAs 0.15x BCAAs | Interm. AAs control | Interm. AAs 0.15x BCAAs | High AAs control | HighAAs 0.15x BCAAs |
|  |  |  |  |  |  |  |  |
|  | median (d) | 77.5 | 75 | 73 | 80.5 | 63.5 | 70 |
|  | maximum (d) | 80.5 | 80.5 | 80.5 | 84 | 70.0 | 77.5 |
|  | Low AAs control |  | 0.0993609 | 0.0062931 | 0.48192167 | 5.917E-17 | 7.5794E-09 |
|  | Low AAs 0.15xBCAAs |  |  | 0.2281607 | 0.28858679 | 1.666E-13 | 3.085E-06 |
|  | Interm.AAs control |  |  |  | 0.0160881 | 2.045E-10 | 0.00054979 |
|  | Interm. AAs 0.15xBCAAs |  |  |  |  | 3.36E-19 | 4.4346E-10 |
|  | High AAs control |  |  |  |  |  | 0.00052954 |
|  | High AAs 0.15xBCAAs |  |  |  |  |  |  |
|  |  |  |  |  |  |  |  |
|  |  |  |  |  |  |  |  |
|  |  |  |  |  |  |  |  |
|  | **Cox Proportional Hazard (CPH) analysis** | | |  |  |  |  |
|  |  |  |  |  |  |  |  |
|  | Number of events |  | 1097 |  |  |  |  |
|  | Number of censoring |  | 78 |  |  |  |  |
|  | Total number |  | 1175 |  |  |  |  |
|  |  |  |  |  |  |  |  |
|  | **Risk Ratios** |  |  |  |  |  |  |
|  |  | Level 1 | /Level 2 | Risk Ratio | p | Lower 95% | Upper 95% |
|  | AA concentration | Interm. | Low | 1.0806773 | 0.2968 | 0.9340671 | 1.250384 |
|  |  | High | Low | 1.9726573 | <0.0001 | 1.698484 | 2.2914517 |
|  |  | High | Interm. | 1.8253898 | <0.0001 | 1.5756584 | 2.11447327 |
|  |  | Low | Interm. | 0.9253456 | 0.2968 | 0.7997543 | 1.0705869 |
|  |  | Low | High | 0.5069304 | <0.0001 | 0.4364046 | 0.5887603 |
|  |  | Interm. | High | 0.5478282 | <0.0001 | 0.472873 | 0.6346553 |
|  | BCAA restriction | 0.15xBCAA | control | 0.8845354 | 0.0427 | 0.7855432 | 0.9959742 |
|  |  | control | 0.15x BCAAs | 1.1305371 | 0.0427 | 1.004042 | 1.273004 |
|  |  |  |  |  |  |  |  |
|  |  |  |  |  |  |  |  |
|  | **Parameter Estimates** |  |  |  |  |  |  |
|  | Term | Estimate | SE | p |  |  |  |
|  | AA conc. | -0.2523232 | 0.0435814 | <0.0001 |  |  |  |
|  | BCAA-restriction | 0.061346 | 0.0302655 | 0.0427 |  |  |  |
|  | AA conc.*BCAA-restriction | -0.1480293 | 0.0430451 | 0.002 |  |  |  |
|  |  |  |  |  |  |  |  |

Table S6 **Statistical analysis for Figure S4**

|  | Survival under starvation BCAA restriction on varying amino acid concentrations | | | | | |  |
| --- | --- | --- | --- | --- | --- | --- | --- |
|  |  |  |  |  |  |  |  |
|  | **p-value (log rank)** | LowAAs control | LowAAs 0.15x BCAAs | Interm. AAs control | Interm.AAs 0.15x BCAA | HighAAs control | HighAAs 0.15xBCAAs |
|  |  |  |  |  |  |  |  |
|  | median (d) | 7.6 | 8.6 | 6 | 8.3 | 5.6 | 6.6 |
|  | mean (d) | 7.7 | 9.0 | 6.6 | 8.5 | 6.1 | 7.2 |
|  | maximum (d) | 9.9 | 9.9 | 9.2 | 11.2 | 7.9 | 9.9 |
|  | Low AAs control |  | 2.11E-05 | 3.772E-06 | 0.0017643 | 1.19E-14 | 0.00470578 |
|  | Low AAs 0.15xBCAAs |  |  | 8.064E-18 | 0.1829419 | 1.97E-29 | 2.5847E-12 |
|  | Interm.AAs control |  |  |  | 1.542E-14 | 0.008583 | 0.02903741 |
|  | Interm.AAs 0.15xBCAAs |  |  |  |  | 4.57E-27 | 1.1497E-09 |
|  | High AAs control |  |  |  |  |  | 4.6885E-08 |
|  | High AAs 0.15xBCAAs |  |  |  |  |  |  |
|  |  |  |  |  |  |  |  |
|  |  |  |  |  |  |  |  |
|  | **Cox Proportional Hazard (CPH) analysis** | | | |  |  |  |
|  |  |  |  |  |  |  |  |
|  | Number of events |  | 394 |  |  |  |  |
|  | Number of censoring |  | 0 |  |  |  |  |
|  | Total number |  | 394 |  |  |  |  |
|  |  |  |  |  |  |  |  |
|  | **Risk Ratios** |  |  |  |  |  |  |
|  |  | Level 1 | /Level 2 | Risk Ratio | p | Lower 95% | Upper 95% |
|  | for AA concentration | Interm. | Low | 1.5172168 | <0.0001 | 1.242343 | 1.8528182 |
|  |  | Low | Interm. | 0.6591016 | <0.0001 | 0.539718 | 0.8049307 |
|  | for BCAA-restriction | 0.15xBCAAs | control | 0.4525444 | <0.0001 | 0.369393 | 0.5543605 |
|  |  | control | 0.15x BCAAs | 2.2097281 | <0.0001 | 1.80388 | 2.707143 |
|  |  |  |  |  |  |  |  |
|  |  |  |  |  |  |  |  |
|  |  |  |  |  |  |  |  |
|  | **Parameter Estimates** |  |  |  |  |  |  |
|  | Term | Estimate | SE | p |  |  |  |
|  | AA conc. | -0.2084388 | 0.050944 | <0.0001 |  |  |  |
|  | BCAA-restr. | 0.3964347 | 0.051743 | <0.0001 |  |  |  |
|  | AA conc.*BCAA-restr. | -0.1394651 | 0.050803 | 0.0061 |  |  |  |
|  |  |  |  |  |  |  |  |
